# Prostate Cancer Theranostics - An Overview

**DOI:** 10.3389/fonc.2020.00884

**Published:** 2020-06-05

**Authors:** Diane Abou, Nadia Benabdallah, Wen Jiang, Lu Peng, Hanwen Zhang, Alexandria Villmer, Mark S. Longtine, Daniel L. J. Thorek

**Affiliations:** ^1^Department of Radiology, Mallinckrodt Institute of Radiology, Washington University School of Medicine, St. Louis, MO, United States; ^2^Program in Quantitative Molecular Therapeutics, Washington University School of Medicine, St. Louis, MO, United States; ^3^Radiology Cyclotron Facility, Mallinckrodt Institute of Radiology, Washington University in St. Louis, St. Louis, MO, United States; ^4^Department of Biomedical Engineering, Johns Hopkins University, Baltimore, MD, United States; ^5^Department of Biomedical Engineering, Washington University in St. Louis, St. Louis, MO, United States; ^6^Oncologic Imaging Program, Siteman Cancer Center, Washington University School of Medicine, St. Louis, MO, United States

**Keywords:** peptide, radionuclide, radiotherapy, alpha particle, prostate cancer

## Abstract

Metastatic prostate cancer is incurable, and novel methods to detect the disease earlier and to direct definitive treatment are needed. Molecularly specific tools to localize diagnostic and cytotoxic radionuclide payloads to cancer cells and the surrounding microenvironment are recognized as a critical component of new approaches to combat this disease. The implementation of theranostic approaches to characterize and personalize patient management is beginning to be realized for prostate cancer patients. This review article summarized clinically translated approaches to detect, characterize, and treat disease in this rapidly expanding field.

## Introduction

Prostate cancer (PCa) is the most common malignancy in men. In 2019 there were ~175,000 new cases of PC with >30,000 deaths, yielding enormous personal, societal, and economic costs (1). For many low-risk patients with primary PCa, “active surveillance” to monitor indolent disease by serial biopsy and prostate specific antigen (PSA) measures is an appropriate option. If treatment is desired for primary PCa, standards of care may involve surgical resection, external beam or proton radiotherapy, and brachytherapy and often are curative. For patients diagnosed with primary PCa, 5-year survival rates exceed 90%. However, for patients with advanced prostate cancer with tumor cells present at distant sites outside of the prostate there are severe impacts on quality of life and a low (<30%) 5-year survival rate. Upon metastasis to the bone, the most common site of PCa metastasis, the 5-year survival rate falls to a dismal 3–5%, making the disease essentially incurable and the second leading cause of cancer death in men (2–6). Conventional treatments for later-stage and metastatic disease can involve anti-hormonal therapies, chemotherapies, further use of radiation, and the use of bone-targeted agents.

An emerging area with significant potential to combat this lethal disease is the use of theranostic agents that detect PCa with exquisite sensitivity and can precisely ablate these sites. The implementation of an expanding array of nuclear medicine approaches to accurately characterize disease status enabling personalized patient management for prostate cancer patients is beginning to be realized. Advances in radionuclide production and availability, chemical synthesis, and clinical trial implementation are rapid and ongoing, and new tools and approaches will undoubtedly emerge in the near future.

**Figure 1:**
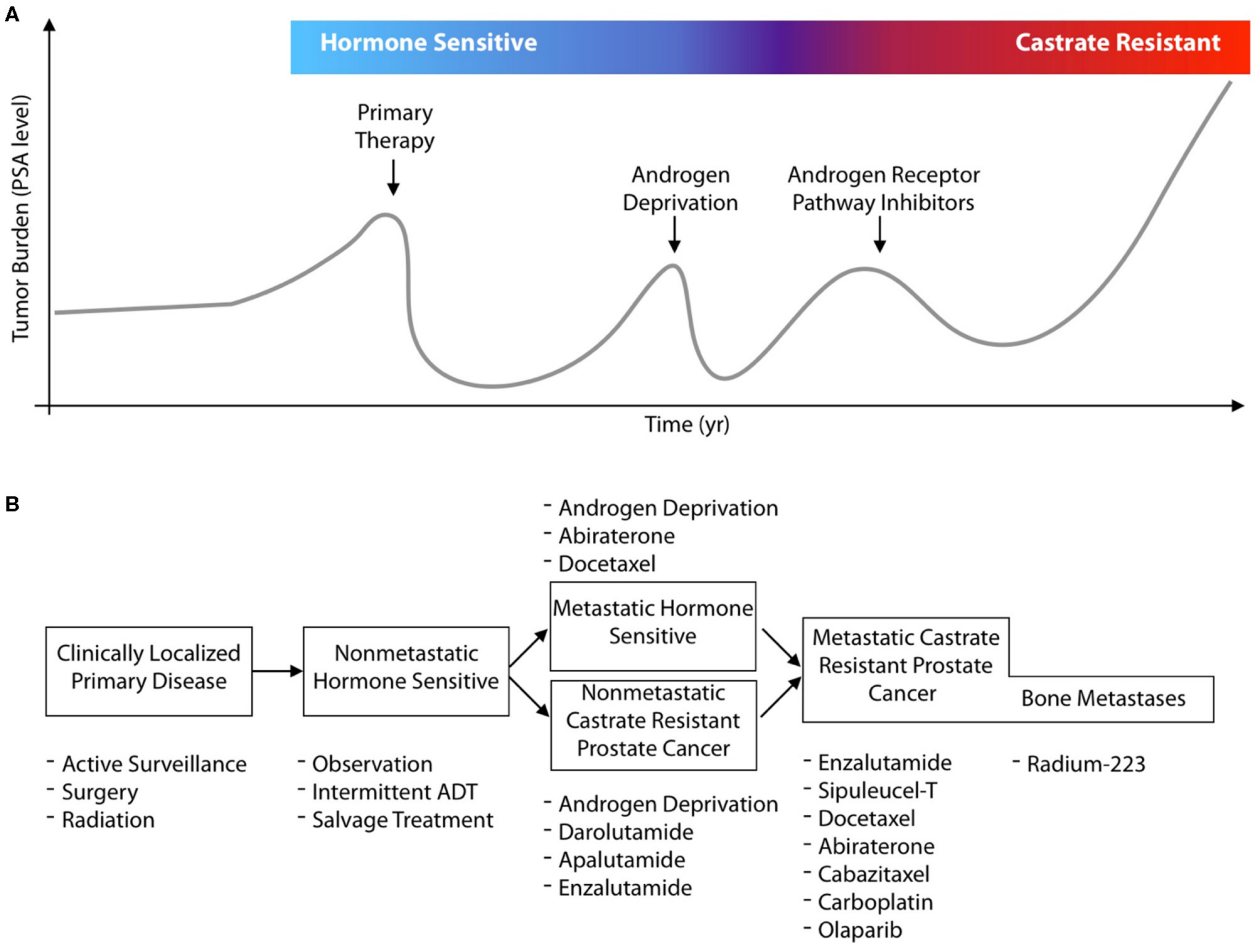
Contemporary prostate cancer states and systemic treatments. **(A)** Adenocarcinoma arising in the prostate gland is the second most common cancer in males, and can be cured with local intervention if detected when still localized to the organ. Disease is often detected by serum PSA measures which also enable tracking of disease recurrence should initial therapy and subsequent lines of treatment fail. Most commonly, disease control is attempted with hormonal control of the androgen receptor signaling access through (chemical) castration and the use of androgen receptor inhibitors. **(B)** The last decade has seen the approval of 8 new agents for prostate cancer across different disease states. These approvals result from significant survival benefits for patients at multiple lines of treatment. However, the eventual progression on novel antiandrogens, Radium-223, and chemotherapies leave considerable room for improvement. Despite the number of existing treatment options, efforts with molecularly targeted radiotherapies are under intense, global, evaluation; as well as to use new imaging agents to better guide drug development and more accurately characterize disease.

In this review, we concisely present molecular imaging and theranostic tools that have been developed to better delineate, monitor, and treat prostate cancer, with a focus on clinically implemented radionuclide theranostics. There have been numerous changes in the management for prostate cancer patients and the options for treatment in the previous decade. In Figure 1A, a general schematic for the progression of disease in a patient is presented, by following their hypothetical disease burden as measured by their secreted PSA (prostate specific antigen) values. Focal treatment options are available for primary disease sites, followed by different systemic treatments at different disease states (Figure 1B). Recent approvals of several different pharmacological and radiological entities by the FDA/EMA, often for the same indications, underline the value for molecularly targeted imaging and therapeutics to guide and enhance patient outcomes.

The present review helps provide context and an overview of the new options and methods leveraged to detect, characterize, and combat disease in this evolving clinical landscape. First, we describe methods using molecular imaging tools for the central oncological driver of the disease, the androgen receptor, to read out pharmacological properties of candidate hormonal therapy. Next, we describe efforts to target radiopharmaceuticals to receptors overexpressed on the surface of prostate cancer cells. Finally, we discuss efforts to target imaging and to direct treatment to the microenvironment of the bone-tropic metastases of this disease.

## Androgen Receptor Imaging With [^18^F]-FDHT

The androgen receptor (AR), an intracellular DNA-binding, hormone-responsive transcription factor, is the key molecular driver for male organ development and is the oncological driver of PCa (7). Activated by binding androgens such as testosterone in the cytoplasm, the AR then translocates to the nucleus and stimulates the expression of genes involved in differentiation and proliferation (8). The effectiveness of repressing this central AR pathway by androgen-deprivation therapy was discovered by Huggins over 70-years ago (9, 10), and remains a mainstay of PCa treatment. However, after an initial response, AR pathway reactivation inevitably occurs, leading to disease progression. Recently developed, highly potent anti-androgen molecules can be employed to some effect, even in late-stage, castrate-resistant PCa [CRPC (11)]. This demonstrates that AR-signaling maintains its central role over the entire course of disease progression (9, 12), rather than CRPC becoming AR-signaling independent. Mechanisms of resistance to anti-androgen therapy in CRPC include AR-receptor gene amplification, AR-upregulation, local hormone production, and/or constitutively active AR-mutations (13–15). Counter intuitively, the term “castrate-resistant” most often reflects continued androgen dependence, rather than evolved AR-pathway independence. Thus, even in CRPC disease, the AR pathway thus remains an appropriate therapeutic and imaging target.

**Figure 2:**
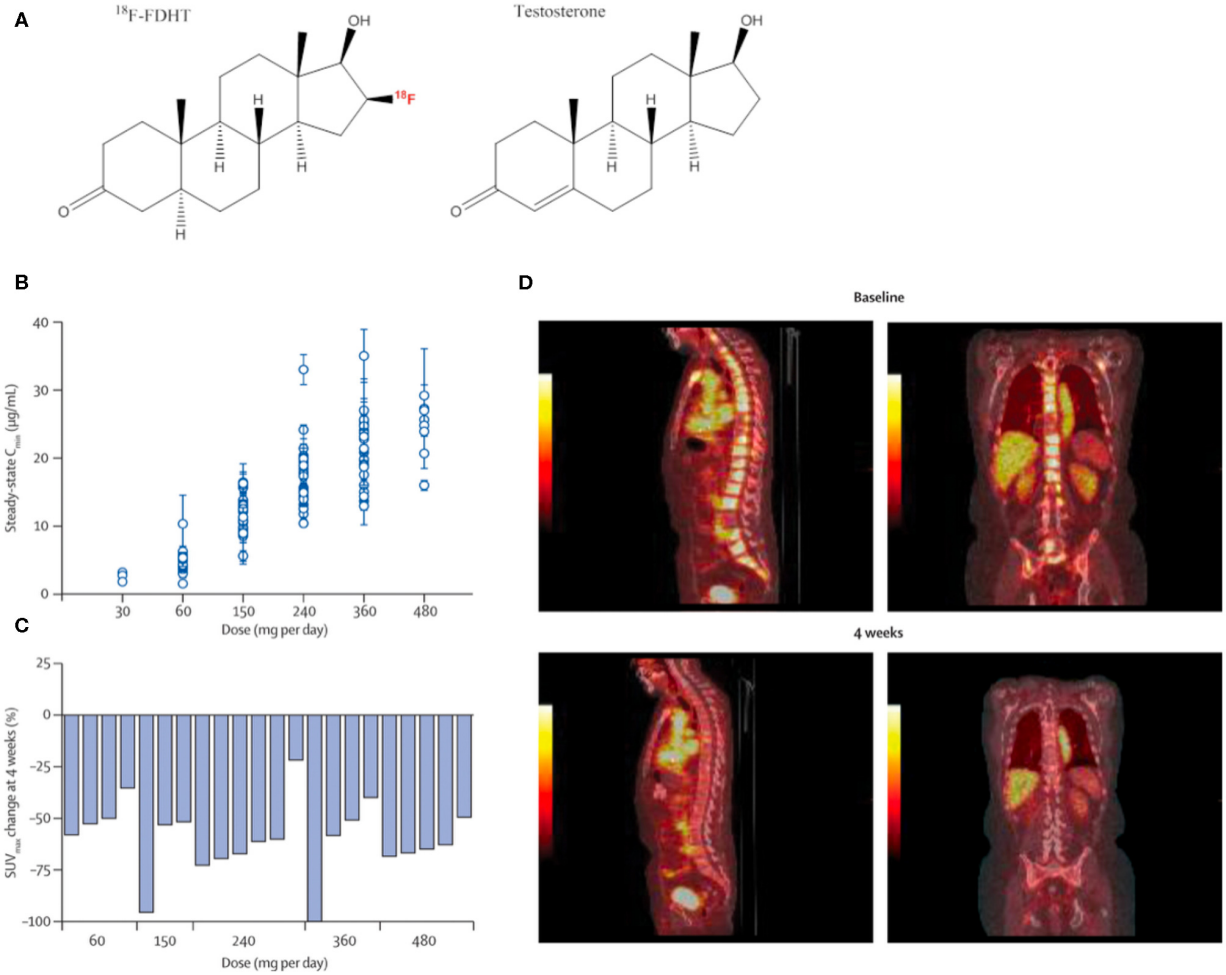
Quantitative imaging of the androgen receptor. [^18^F]-FDHT, which annotates the AR, the central molecular driver of prostate cancer development and progression, is an effective imaging tool to evaluate pharmacodynamic features of candidate therapies. MDV3100 treatment, a second-generation orally bioavailable anti-androgen, was tested in men with castrate-resistant prostate cancer. **(A)** Chemical structure of positron-emitting ^18^F-FDHT and testosterone. **(B)** MDV3100 serum concentration by dose level. **(C)** Representative baseline and treatment scans showing marked decrease in radio-androgen uptake in skeletal metastases. Reproduced from Scher et al. (16) **(D)** Change in standardized uptake value of [^18^F]-FDHT PET for men at baseline compared to 4 weeks of treatment.

16β-[^18^F]-fluoro-5α-dihydrotestosterone ([^18^F]-FDHT) is a positron-emitting analog of the native AR-binding dihydrotestosterone with a conjugated positron-emitting radionuclide ([^18^F]; Figure 2A) and has been used in small animals studies (17, 18) and in clinical studies to evaluate AR-expression levels and occupancy (19–21). [^18^F]-FDHT positron emission tomography (PET) enables detection of metastatic lesions, as indicated by increased concentrations of AR, and is being evaluated for its capacity to phenotype lesions in concert with other conventional imaging modalities (22).

The majority of CRPC escape anti-androgen therapy by AR-signaling amplification, and hormonal therapies that can inhibit AR-signaling are a mainstay of treatment for CRPC. Thus, imaging the expression levels of AR is a viable strategy to measure receptor density and the pharmacological response to these anti-androgen therapies (22, 23) (Figures 2B–D). These features have been exploited in the clinical evaluation of next generation anti-androgens in early clinical trial to directly quantitate AR-blockade. Reduction in [^18^F]-FDHT uptake and a plateau, consistent with saturation of AR binding, can be quantitated directly on a lesional or patient basis (16, 24).

## Prostate Specific Membrane Antigen Imaging

Prostate Specific Membrane Antigen (PSMA) has emerged as the pre-eminent prostate cancer target for diagnostic imaging, assisting efforts to detect disease earlier, monitor recurrence, and track the progression of disease. PSMA is a robust target for PCa, with numerous pathological studies reporting elevated PSMA expression on 85–100% of prostate cancers (25–28). The number of clinical trials that use PSMA-targeted agents for diagnostic or therapeutic purposes is large and continues to expand: in the clinicaltrials.gov database, there are currently over 100 clinical trials that utilize PSMA for imaging or therapy.

PSMA is a type II transmembrane protein present on the cell surface, and, interestingly, PSMA contains an extracellular domain with glutamate carboxypeptidase activity (29). Although neither the exact physiological role of PSMA nor the reason for its overexpression on PCa cells is known, it has been used utilized in the targeting of multiple PSMA-binding small molecules, many of which are urea based and bind to the enzymatic site. We note that the use of PSMA as nomenclature to refer to this protein can be confusing, as this receptor is expressed in multiple non-prostate-derived tissues including the brain, peripheral nerves, salivary glands, gut, and kidney. Due to its independent discovery in multiple tissues, this protein, which we will refer to as PSMA, is also identified in the literature as glutamate carboxypeptidase II (GCPII) in the gut and as N-acetyl-L-aspartyl-L-glutamate peptidase I of NAAG peptidase I (NAALADase I) in the brain.

**Figure 3:**
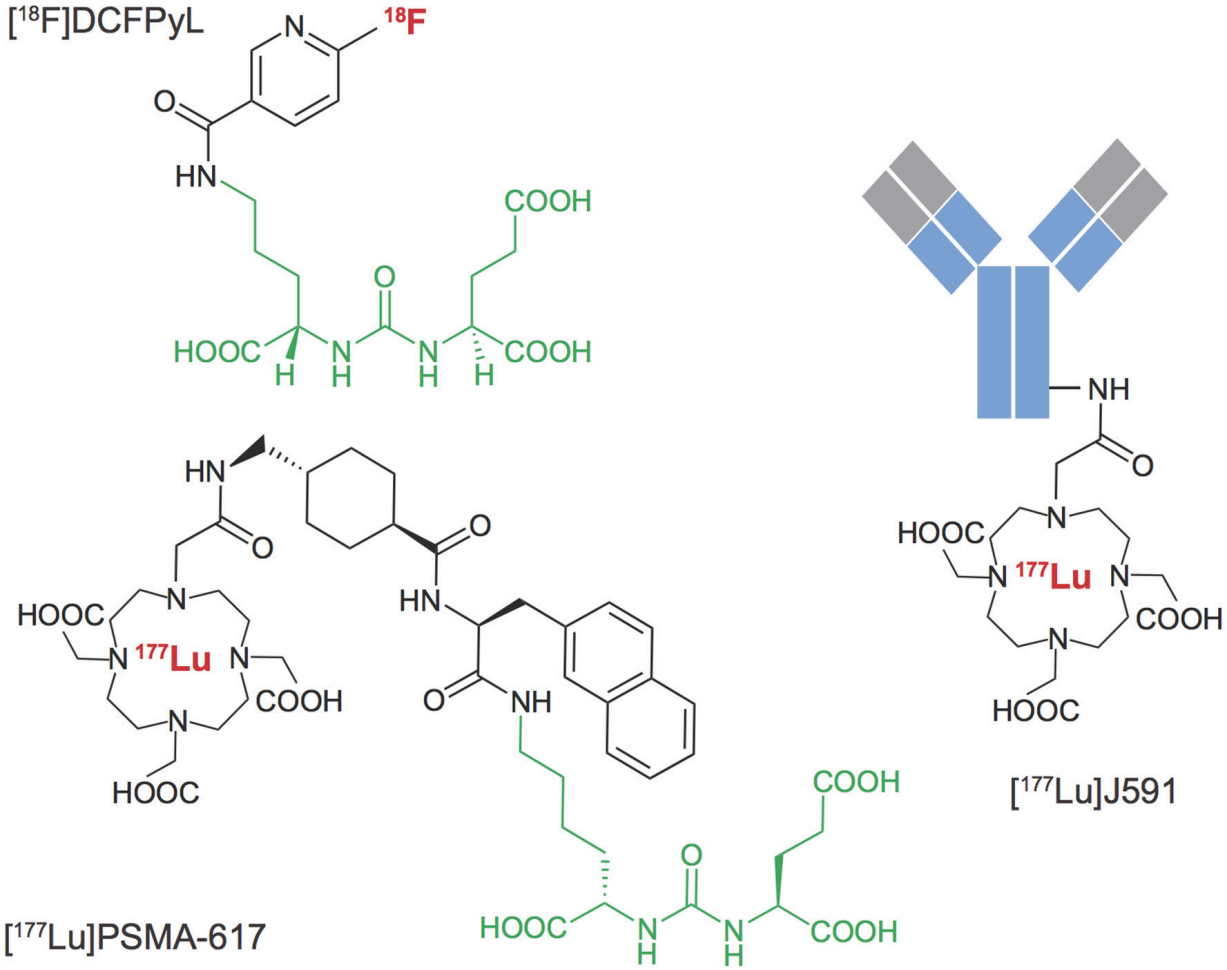
PSMA Targeted radiopharmaceuticals. Examples of diagnostic and therapeutic agents targeting Prostate Specific Membrane Antigen. A common urea-based motif (green) has been widely derivatized for localization at sites of prostate cancer metastases that over-express the membrane-localized PSMA metalloenzyme. Antibody targeting vectors with high affinity for PSMA have also been labeled with positron, beta-particle and alpha-particle emitting radionuclides.

An array of agents have been developed that target PSMA with high affinity, including antibodies and small molecules. PSMA imaging agents for use in PET imaging and single-photon emitting computed tomography (SPECT) have been evaluated in preclinical and clinical settings, using an array of positron emitting radionuclides such as Fluorine-18, Gallium-68, Scandium-44 and Zirconium-89, or single-photon emitting radionuclides such as Technetium-99m and Iodine-125. Examples of widely tested structures and antibodies are found in Figure 3. Multiple PSMA imaging agents have been tested in humans and several have been evaluated in late stage, multicenter, clinical trials (Table 1).

**Table 1:** Selected prostate cancer trials for disease detection and radiotherapy treatment of metastatic disease.

**CTID**	**Abbreviated titles**	**Purpose**	**Phase**	**Compound**	**Patients**	**Status**
NCT02981368	18F-DCFPyL PET/CT imaging in patients with prostate cancer (OSPREY)	Diagnostic	II/III	[18F]-DCFPyL	385	Completed
NCT03739684	18F-DCFPyL PET/CT imaging in patients with suspected recurrence of prostate cancer (CONDOR)	Diagnostic	III	[18F]-DCFPyL	200	Active
NCT03392428	177Lu-PSMA617 theranostic Vs. cabazitaxel in progressive metastatic CRPC (TheraP)	Therapy	III	[177Lu]-PSMA-617	201	Active
NCT03511664	177Lu-PSMA-617 in metastatic castrate-resistant prostate cancer (VISION)	Therapy	III	[177Lu]-PSMA-617	750	Active
NCT03276572	225Ac–J591 in patients with mCRPC	Therapy	Ib	[225Ac]-J591	42	Recruiting
NCT03939689	I-131-1095 radiotherapy in combination with enzalutamide in patients with mCRPC (ARROW)	Therapy	II	[131I]-MIP-1095	175	Recruiting
NCT02552394	Radioimmunotheraspy in prostate cancer using 177Lu-J5912 antibody	Therapy	I	[177Lu]-J591	54	Recruiting

The first effort to target and image PSMA involved the 7E11-C5.3 murine antibody, which targets an intracellular epitope of PSMA (30, 31). Labeled with Indium-111, permitting imaging by SPECT, this agent was known as capromab pendetide or ProstaScint and was approved in 1996 for the detection of PCa lesions (32, 33). This initial PSMA-targeting agent found limited clinical implementation due to low contrast imaging, likely resulting from low accessibility *in vivo* to the intracellular PSMA epitope, concerns over human anti-murine immune responses, and competing conventional imaging methods.

A substantial improvement in PSMA imaging was achieved with the generation of J591, a humanized antibody targeting an epitope on the extracellular region of PSMA. J591 has been labeled with radionuclides for both PET and SPECT imaging of PCa lesions in humans (34–36). However, as antibody and antibody-fragment derived agents require administration of the radiolabeled agent several days before effective PSMA/tumor imaging can be performed, as this time is required for clearance of the tracer and optimal tumor/background signal. This relatively longer time frame from administration to imaging, which requires a return visit of the patient, has reduced enthusiasm for J591-based PSMA imaging.

Instead, much of the current intense interest in PCa-lesion imaging using PSMA revolves around PSMA-targeting small molecules (37). Small molecules can have exquisite targeting sensitivity and specificity, and they allow a much more rapid turnaround from agent injection to imaging (minutes to hours) than do antibodies (days). Also, small molecule agents that rapidly target PSMA allow the use of short-lived PET radionuclides, permitting high resolution, and sensitive detection with a reduced radiation dosed compared to antibody-based PET imaging methods (38, 39).

**Figure 4:**
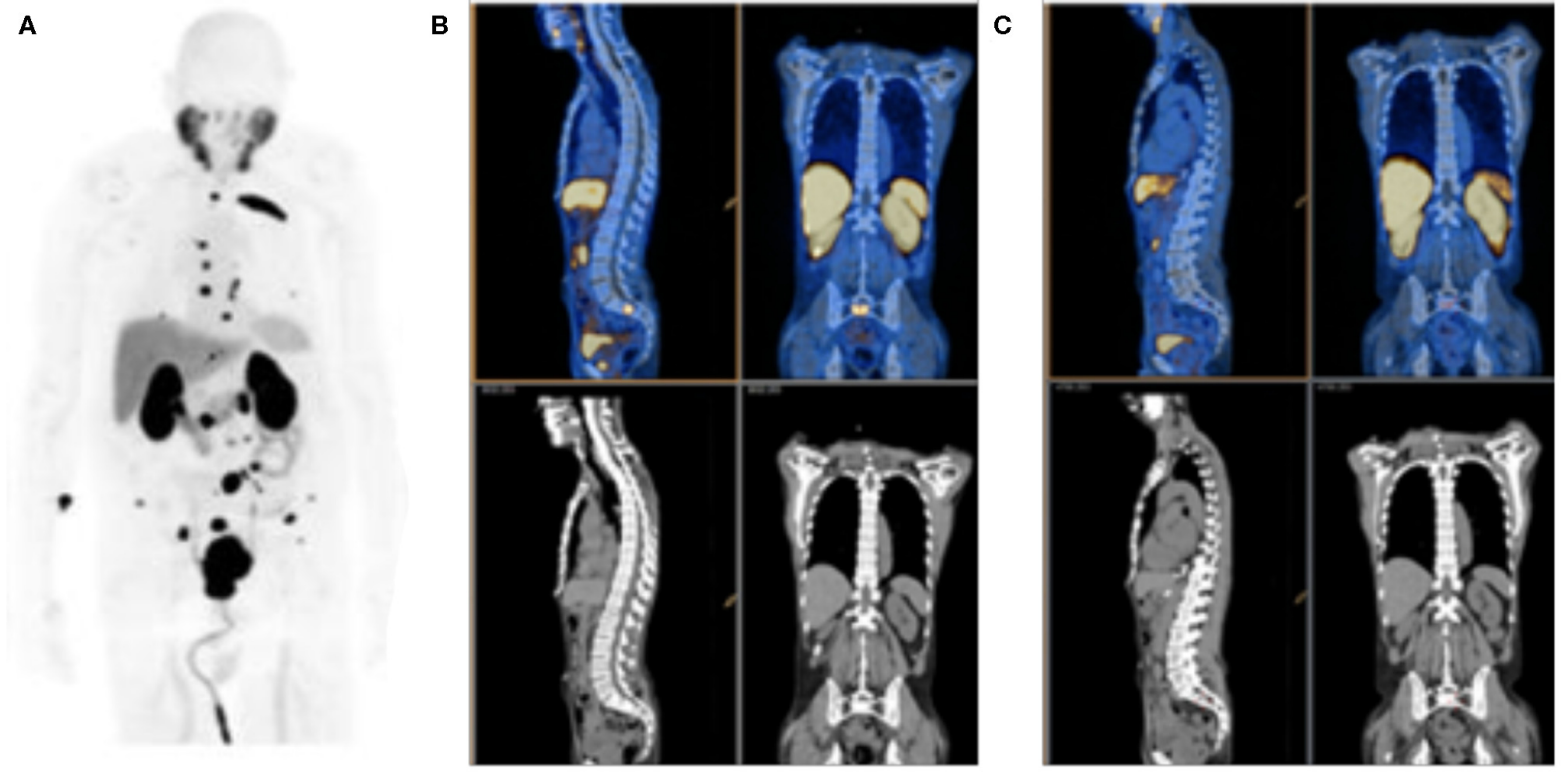
PSMA PET Imaging. **(A)** Detection of prostate cancer lesions with [^18^F]DCFPyL, along with uptake in kidneys, salivary gland and liver. **(B)** Pre-treatment [^68^Ga]PSMA-11 and **(C)** post-treatment PET/CT, top, and CT, bottom, scans obtained on day 9 after initiating combined anti-androgen hormone therapy. Reproduced from **(A)**(40) and **(B,C)** (41).

A large number of PSMA-targeted small molecules have been tested for PCa imaging in preclinical models and in humans. An example of a patient scan with the high affinity fluorinated agent is shown in Figure 4A. Multiple prospective clinical trials with targeted small-molecule PSMA imaging agents are underway, with encouraging results on the sensitivity of detection and the utility of these tracers in clinical settings (42–44). The most widely reported clinical imaging has been performed with PET imaging using radiometal-labeled [^68^Ga]PSMA 617 or [^68^Ga]PSMA-11 or using fluorinated [^18^F]-DCFPyL (45). There are currently many options for radionuclide and PSMA-targeting ligand, and several studies to compare imaging features have been undertaken. Optimal uptake time, magnitude of tumor and background organ uptake, and imaging resolution are dependent on ligand and radionuclide. Logistical and cost issues are also a consideration. Multiple doses of Fluorine-18 radiolabeled inhibitor are available at institutions with a medical cyclotron, with the ability to ship agent at greater distances. Lower upfront costs are required for Gallium-68 isolation on a ^68^Ge/^68^Ga generator system, however fewer doses are produced with lower specific activity, and decreased resolution (46–49).

The capacity to perform highly sensitive molecular imaging of PSMA to detect minute foci of metastatic disease, led by PET-isotope labeled, PSMA-targeted small molecules, is motivating a paradigm shift in prostate cancer patient management. For example, the ability to rapidly determine lesion response on a given treatment regimen (41, 50, 51), as shown in Figures 4B,C, or to target external beam radiotherapy to sites of oligometastatic disease (52, 53), are means of precise disease control not previously possible. Of course, it should be noted that rigorous, prospective, controlled and multi-center trials, and statistical analyses are required before we can be confident that these new tools provide real-world benefit for patients.

## Prostate Specific Membrane Antigen Targeted Radiotherapy

Concurrent with the development of the PSMA-targeted diagnostic agents described above, the application of PSMA-directed targeted radiotherapy of PCa is an ongoing area of great potential. Here, PSMA-binding ligands are labeled with radionuclides that produce potent cytotoxic decay products, without or with the co-emission of imageable photons. Both beta-particle (Lutetium-177, Copper-67, Iodine-131) and alpha-particle (Bismuth-213 and Actinium-225) emitting PSMA-targeted agents are at various stages of drug development (54–58). It is noteworthy that PSMA-ligands are internalized by endocytosis after PSMA binding, allowing increased intracellular levels of residualizing therapeutic radionuclides and improved potential for tumor-cell killing. Several PSMA-targeted radiotherapeutic agents have begun to be applied in late stage metastatic prostate cancer (PCa) patients (58–62); Table 1.

The most developed agent in the PSMA-targeted radiotherapeutic class is [^177^Lu]PSMA-617. [^177^Lu]PSMA-617 is inherently theranostic in that the decay pathway of ^177^Lu emits both tumor-cell-killing beta particles and imageable photons, detectable by planar scintography, and SPECT imaging. Thus, [^177^Lu]PSMA-617 allows both therapy and imaging of agent distribution and uptake with the same agent. The majority of response data for this agent have been accrued from retrospective trials that have shown efficacy in reducing PSA levels with manageable hematological, renal, and salivary gland toxicity (63–69). Patient characteristics varied to a large degree with respect to previous treatment, disease stage, and radiographic and biochemical features (70). Thus, these results are encouraging as they indicate that there may be a benefit for patients along the spectrum of disease burden and stage, but also present a problem as informing to how these agents can be wielded most effectively. To aid in answering these questions, well-powered prospective trials with [^177^Lu]PSMA-617 are now underway.

**Figure 5:**
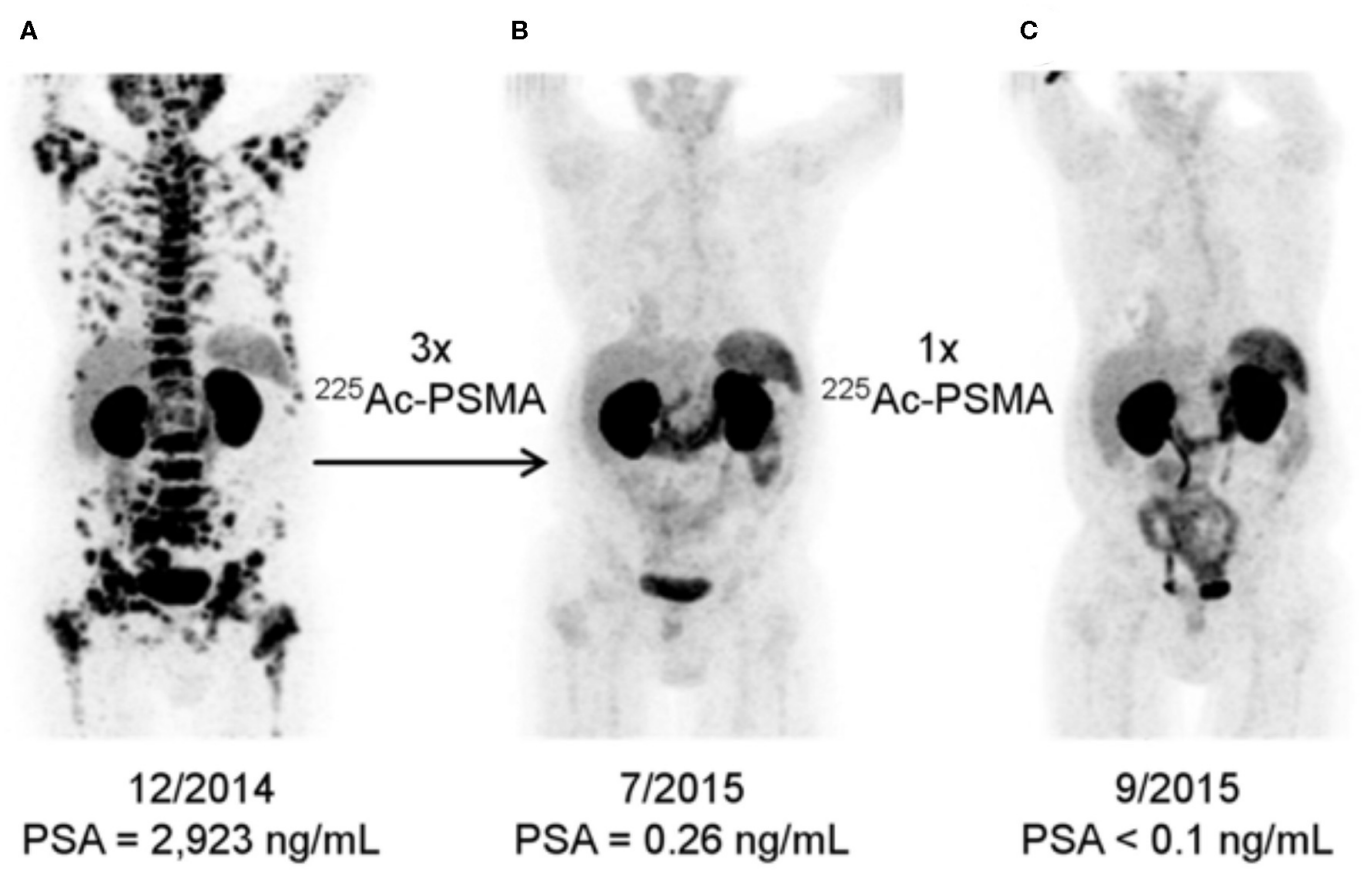
[^68^Ga]PSMA-11 Imaging Response to [^225^Ac]PSMA-617. Alpha-particle emitting Ac-225 bound to the DOTA chelator-bearing PSMA-617 scaffold was used to treat a patient following disease progression after several lines of conventional treatment and [^177^Lu]PSMA-617. Serial images during the treatment course reveal significantly less PSMA expression and potential lesion eradication. A decrease in salivary gland uptake is also noted, as it is a site of PSMA expression inadvertently treated by these agents. Reproduced from (71).

Great interest has also been generated by the application of PSMA-617 labeled with ^225^Ac, which emits four cell-killing alpha particles. This agent has been studied in small cohorts of men in Germany and in South Africa and clinical findings have generated great interest, Figure 5 (58, 71, 72). Alpha particles emitted from heavy isotopes, such as Actinium-225 and Radium-223 (described below), have high energies, in the 5-8 MeV range, that produce extremely cytotoxic genomic damage. Alpha particles also exhibit a much shorter path length than β-particles, such as those emitted by Lutetium-177. Together, the radiobiological properties of alpha-particle emitting agents mean that even small deposits of cancer cells could be eradicated with appropriate uptake (57), while largely sparing adjacent and distant tissues because of their short path length. Investigations with additional alpha-particle emitting theranostics, including with generator-produced [^213^Bi]PSMA-617 and [^212^Pb]PSMA-617 (57, 73), are also being evaluated, which may alleviate sourcing issues regarding ^225^Ac.

However, the expression of PSMA on non-prostate-derived cells as noted above leads to a major concern in the application of PSMA-targeted therapies: the on-target but off-tumor localization. Dosimetric evaluation of the distribution of the diagnostic and theranostic ligands provides a means to predict absorbed doses to sites of both healthy and diseased tissue uptake. Radiosensitive organs, such as the hematopoietic niche of the bone marrow, kidneys, nerves, and intestine often account for the dose-limiting activity that can safely be administered to a patient (74, 75). The major side-effect producing sites of undesired localization of PSMA-targeted agents identified at this time include the salivary glands and kidney, which highly express PSMA, are radiosensitive and are critical for survival and quality of life. Xerostomia from non-repairable damage to the salivary glands has been reported with ^225^Ac-PSMA-617, with varying degrees of glandular damage observed with ^177^Lu-PSMA-617. Interestingly, biological targeting vectors such as ^177^Lu-J591 which do not apparently accumulate in the salivary glands may have additional utility with the potential for a favoragle therapeutic window (76, 77). The much longer-onset effect of severe kidney toxicity has not yet been clinically noted (63). However, studies to date generally lack rigorous patient enrollment and follow-up criteria and have not followed patients for long periods of time. Prospective and randomized trials will be needed to compare patient benefit against conventional treatments and the effectiveness of means to reduce these off-tumor toxicities.

## GRPR Bombesin

The gastrin-releasing peptide receptor (GRPR) is expressed on a wide range of cell types in higher mammals, especially in the nervous system and gastrointesinal tract (78, 79). Small peptides interact with GPRP to modulate a wide range of cell and organ functions (80). GRPR is aberrantly overexpressed on the cell surface of many cancers, including lung, breast, and prostate cancer (81, 82). The bombesin subfamily is the best studied GRPR, and a large number of mammalian and amphibian bombesin peptide analogs have been radiolabeled for cancer imaging and therapy (82).

Because of the frequent overexpression of GRPR in prostate cancer, several bombesin radiopharmaceuticals have been tested in humans for PCa disease detection, including [^68^Ga]RM2, [^68^Ga]BAY86-7548, and [^64^Cu]-CB-TE2A-AR06, among others. Studies reveal high-contrast detection of disseminated and primary disease (83–86). While GRPR expression may not be as ubiquitous on prostate cancer cells as PSMA, there is no background target expression in the kidneys or salivary glands. Thus, there may be utility for other prostate cancer targeted theranostics in addition to PSMA (87), especially in the GRPR class.

## Theranostics for the Bone Metastasic Microenvironment

The skeletal compartment is the most frequent site of metastases in prostate cancer patients. These lesions are often painful, and may also further degrade quality of life through fracture, spinal cord compression, hypercalcemia, and impaired mobility (3, 88). Bone metastases occupy a nutrient-rich niche that enhances the treatment-resistant nature of disseminated tumor cells (89). Early detection and specific localized treatment of these disseminated sites are recognized as necessary components of a successful strategy to combat bone metastatic prostate cancer.

Conventional imaging modalities for PCa bone metastases include magnetic resonance and X-ray computed tomography, which are commonly applied in concert with nuclear medicine scans for accurate bone lesion detection. [^99m^Tc]-bisphosphonates and [^18^F]-NaF are both bone-seeking agents that are incorporated at sites of active bone remodeling adjacent to metastatic foci and are used for imaging. Approved beta particle-emitting agents for bone pain palliation are [^89^Sr]chloride and [^153^Sm]EDTMP, an ion and phosphonic acid ligand, respectively, which are taken up at or near sites of bone metastasis. Both produce imageable emissions for planar imaging in order to evaluate uptake. Unfortunately, the long path length of these energetic beta particle emissions result in irradiation of the bone marrow, a dose limiting organ, and these agents have not produce survival improvements when evaluated in clinical trial.

The first bone-targeted radionuclide that aids in pain palliation and also achieves an overall survival benefit over standard of care is the alpha-particle emitting [^223^Ra]Cl_2_ citrate, tradename Xofigo (90). Radium-223 is a calcium-mimetic and localizes to sites of active bone turnover, where it subsequently decays, irradiating nearby prostate cancer cells. The short path lengths of the alpha particles do not result in anemic responses and the drug is well-tolerated. While difficult to image, efforts are underway to provide quantitative assessment of ^223^RaCl_2_ distribution to inform absorbed dose measures at sites of disease and background organs (91–94).

## Conclusion

Agents that specifically and sensitively target molecular features of prostate cancer are being brought to bear to detect, guide, and deliver treatments for men with advanced prostate cancer. The underlying initiator of PCa, the androgen receptor, is the driver of prostate adenocarcinoma development, can be visualized with [^18^F]-FDHT. This imaging tool can evaluate the pharmacological impact of anti-androgens and their efficacy. Cell surface antigen targeted agents, in particular PSMA targeted urea-based ligands and antibodies, have now been assessed in a wide range of scenarios to detect and treat metastatic prostate cancer. Prospective clinical trials that are currently recruiting or underway will provide clearer information on the utility of these new theranostic approaches to improve quality of life and overall survival. These agents have the capacity to deliver ablative doses to sites of disease throughout the body with the potential to overcome this currently incurable disease. Differences in the imaging properties and therapeutic niche for different molecular entities and radionuclides are of continuing research interest to provide optimal patient-specific diagnostic information and therapeutic outcome. These ongoing trials may also shed light on the important question of how these novel imaging and therapy agents will integrate with current treatment modalities and approved imaging methods, including conventional imaging and imaging of prostate cancer cell metabolism with established agents such as [^11^C/^18^F]-choline, [^18^F]-fluorodeoxyglucose and [^18^F]-fluciclovine.

## Author Contributions

All authors listed have made a substantial, direct and intellectual contribution to the work, and approved it for publication.

## Conflict of Interest

The authors declare that the research was conducted in the absence of any commercial or financial relationships that could be construed as a potential conflict of interest.
